# Supplementary material for: Resolving the full spectrum of human genome variation using Linked-Reads
Source: Genome Res. 2019 Apr;29(4):635–45. doi: 10.1101/gr.234443.118 (PMC6442396; doi:10.1101/gr.234443.118)
Supplement: Supplemental Material [file supp_gr.234443.118_Supplemental_longranger-2.2.zip › longranger-2.2.tar.bz2!/lr2.2-public/lib/stan/cmdstan/stan/doc/api/html/agrad_2rev_2functions_2if__else_8hpp_source.html]

Stan: src/stan/agrad/rev/functions/if\_else.hpp Source File


|  |  |
| --- | --- |
|  | Stan  2.5.0  probability, sampling & optimization |


- Main Page
- Related Pages
- Namespaces
- Classes
- Files

- File List
- File Members

All Classes Namespaces Files Functions Variables Typedefs Enumerations Enumerator Friends Macros Pages

- src
- stan
- agrad
- rev
- functions

if\_else.hpp

Go to the documentation of this file.

1 #ifndef STAN\_\_AGRAD\_\_REV\_\_FUNCTIONS\_\_IF\_ELSE\_HPP

2 #define STAN\_\_AGRAD\_\_REV\_\_FUNCTIONS\_\_IF\_ELSE\_HPP

3

4 #include <stan/agrad/rev/var.hpp>

5

6 namespace stan {

7  namespace agrad {

8

17  inline var if\_else(bool c, const var& y\_true, const var&y\_false) {

18  return c ? y\_true : y\_false;

19  }

29  inline var if\_else(bool c, double y\_true, const var& y\_false) {

30  if (c)

31  return var(y\_true);

32  else

33  return y\_false;

34  }

44  inline var if\_else(bool c, const var& y\_true, const double y\_false) {

45  if (c)

46  return y\_true;

47  else

48  return var(y\_false);

49  }

50

51  }

52 }

53 #endif

var.hpp

stan::agrad::var

Independent (input) and dependent (output) variables for gradients.

**Definition:** var.hpp:27

stan::agrad::if\_else

var if\_else(bool c, const var &y\_true, const var &y\_false)

If the specified condition is true, return the first variable, otherwise return the second variable...

**Definition:** if\_else.hpp:17


---

[ Stan Home Page ]

*© 2011–2014,
Stan Development Team.*
